# Migrants and refugees: Improving health and well-being in a world on the move

**DOI:** 10.1371/journal.pmed.1002876

**Published:** 2019-07-30

**Authors:** Richard Turner, on behalf of the PLOS Medicine editors

**Affiliations:** Public Library of Science, San Francisco, California, United States of America and Cambridge, United Kingdom

## Abstract

The PLOS Medicine Editors discuss migrant and refugee health, and announce a forthcoming special issue devoted to the topic.

At the 72nd World Health Assembly, held during May 20–28 of this year in Geneva, Switzerland, a very welcome global action plan was agreed upon, which seeks to establish a “framework of priorities and guiding principles… to promote the health of refugees and migrants” [1]. Indicating the pressing need for leadership by WHO and other actors, over the period from 2000 to 2017, the number of international migrants is estimated to have risen by 49%, to 258 million people [2]. The WHO document also notes that the number of forcibly displaced people has reached its highest-ever level, at an estimated 68.5 million individuals, including 25.4 million refugees—the majority hosted in low- and middle-income countries. Furthermore, approximately 10 million stateless people lack basic human rights to freedom of movement, education, and healthcare. Scattered across the planet, such enormous numbers of people dwarf the individual populations of many countries, yet, all too often, no government or international agency can offer adequate protection or health provision to this virtual state of refugees and migrants.

Migration, whether voluntary or otherwise, covers a wide range of situations. People may move within their own country or to another country for economic or family reasons, and, even in familiar surroundings or in transit, individuals will have health needs that may not be addressed, and they can also be vulnerable to exploitation or violence. Where there is armed conflict or persecution, the degree of danger and vulnerability is substantially greater. One distressing example is the Syrian conflict, which has involved extreme and prolonged violence and led to the harm and displacement of large numbers of people since 2011. UNHCR, the UN Refugee Agency, estimates that, as of June 2019, 5.6 million people have been displaced to Turkey, Lebanon, Jordan, and other nearby countries, with 6.6 million people displaced within Syria itself [3]. Such large and unpredictable movements of refugees create great challenges in protection and provision of shelter, food and water, and medical care.

There is substantial documentation of the numerous and grave health threats faced by migrants, refugees, and asylum seekers. Migrant workers who have relocated internationally are at risk of occupational injuries and ill health, for instance, highlighting the need for employers and host country governments to strengthen employment rights and healthcare provision [4]. Migrants and refugees can be vulnerable to serious outbreaks of infectious diseases, such as cholera, in emergency settings [5]. In a transit or destination country, people could be affected by diseases prevalent in their country of origin, such as tuberculosis, and by noncommunicable diseases, for example, that reflect the situation in countries of transit and destination. Mental ill-health, including posttraumatic stress disorder in relevant groups of people, is a particular concern for migrants and refugees and their health providers [6]. In many settings, barriers of language, culture, or law prevent migrants from accessing essential services. As discussed in an article by Cathy Zimmerman and colleagues published as part of the “Migration & Health” Collection in *PLOS Medicine* in 2011, migration can be viewed in terms of distinct phases, from predeparture to potential return to a person’s country of origin, with opportunities for health monitoring and intervention through suitable services at each stage [7].

Some of the drivers of the growing phenomenon of human migration include population expansion, increased availability of long-distance travel, and greater access to economic opportunities for those willing and able to move. Alongside these factors, it would be naïve not to acknowledge the potential impact of migration on the populations and infrastructure of destination countries—witness the heated debate in the United States over its long and tortuous border with Mexico and the people who attempt to cross that divide. Owing to the prominent, large-scale challenges presented in many countries by the fluid and unpredictable nature of migration, regrettable political constituencies have emerged that can marginalize and stigmatize migrants and refugees. Based as they are on the nonsensical idea that one person merits appropriate access to healthcare and other services but another person does not, these political entities are, though dangerous and destructive, vulnerable to those who can mobilize principled and just arguments. Generally, stable governments can be expected to provide the necessary resources and plan the provision of suitable infrastructure and health services for those who have migrated to their jurisdiction, and where they do not, support from international agencies must be made available.

International agreements set out clear responsibilities for protection of and provision for refugees and asylum seekers, whereas the situation for other migrants is less clear—indicating the potential importance of the new WHO plan in driving the activities of host states and appropriate international bodies. Priorities of the WHO plan include deploying public health interventions to improve migrant health alongside promotion of essential health services and occupational health provision. Strengthening health monitoring and information systems is recommended, as is accelerated progress toward universal health coverage. There should be attention toward mainstreaming migrant and refugee health, the plan notes, and to overcoming misconceptions about these groups of people. We look forward to seeing the impact of the plan in practice.

Seeking to raise awareness of the health threats faced by migrants and refugees and to promote research, service, and policy innovation in this area, the editors of *PLOS Medicine* are planning a Special Issue on the topic to be published early in 2020. Paul Spiegel, director of the Johns Hopkins Center for Humanitarian Health, who will be a Guest Editor for the issue, comments that “migration is a global phenomenon that will likely increase due to improved communication and modes of transport as well as, unfortunately, due to climate change. Health is a human right, and we must all work together to provide appropriate health services to migrants that are equitable, affordable and take into account services available to nationals.” A call for papers has been issued separately setting out the detailed scope for the Special Issue, and we look forward to considering your research papers dedicated to understanding and improving the health and well-being of refugees and migrants in all settings.
